# Prevalence and factors associated with violence and abuse of older adults in Mexico’s 2012 National Health and Nutrition Survey

**DOI:** 10.1186/s12939-016-0315-y

**Published:** 2016-02-27

**Authors:** Maria Guadalupe Ruelas-González, María Beatriz Duarte-Gómez, Sergio Flores-Hernández, Doris Veronica Ortega-Altamirano, Jesus David Cortés-Gil, Arianna Taboada, Ana Lorena Ruano

**Affiliations:** Instituo Nacional de Salud Pública, Cuernavaca, México; Centro de Estudios para la Equidad y Gobernanza en los Sistemas de Salud de Guatemala, and Center for International Health, Bergen University, Bergen, Norway; Independent consultant, Quintana Roo, Mexico

**Keywords:** Abuse, Violence, Older adults, Aging, Mexico, Maltrato, Violencia, Personas mayores, México

## Abstract

**Background:**

Factors associated with violence and the abuse of older adults are understudied and its prevalence in Mexico has not been reported. The aim of this study was to identify the prevalence and factors associated with violence and abuse of older adults in Mexico.

**Methods:**

We used Mexico’s 2012 National Health and Nutrition Survey, which included a sample of 8,894 individuals who are 60 years or older and who self-reported a negative health event related to robbery, aggression or violence in the previous 12 months. We used chi-squared test and Fisher’s exact test to analyze the variables related to violence. Adjusted estimates were completed with multiple logistic regression models for complex surveys.

**Results:**

The prevalence of violence was 1.7 % for both men and women. In 95 % of the cases, the aggression was from an unknown party. Verbal aggressions were the most prevalent (60 %). Among men, physical aggression was more common. Violence frequently occurred in the home (37.6 %); however, men were primarily assaulted in public places (42.4 %), in comparison to women (30.7 %). There were also differences in the risk factors for violence. Among men, risk was associated with younger age (60–64 years), higher education (secondary school or above) and higher socioeconomic status. Among women, risk was associated with depression, not being the head of the family, and region of the country.

**Conclusions:**

Violence against older adults presents differently for men and women, which means it is necessary to increase knowledge about the dynamics of the social determinants of violence, particularly in regards to the role of education among men. The relatively low prevalence found in this study may reflect the difficulty and fear of discussing the topic of violence. This may occur because of cultural factors, as well as by the perception of helplessness perpetuated by the scarce access to social programs that ensure protection and problem solving with regards to the complex social determinants of individual and family violence that this population group endures.

## Abstracto

**Antecedentes:**

Los factores asociados con la violencia y el abuso de los adultos mayores están poco estudiados y su prevalencia en México no ha sido reportada. El objetivo de este estudio fue identificar la prevalencia y los factores asociados a la violencia y el abuso de los adultos mayores en México.

**Metodología:**

Se utilizó la Encuesta Nacional de Salud y Nutrición 2012. La muestra estuvo conformada por 8,894 personas de 60 años de edad o mayores que autoreportaron haber sufrido algún daño a la salud por robo, agresión o violencia en los últimos 12 meses. Se efectuó un análisis de factores asociados con la violencia con las pruebas ji cuadrada y la exacta de Fisher; se obtuvieron estimaciones ajustadas con modelos de regresión logística múltiple para encuestas complejas.

**Resultados:**

La prevalencia de violencia fue de 1.7 % para ambos sexos. En el 95 % de los casos, la agresión provino de personas sin vínculo filial. Predominaron las agresiones verbales (60 %), aunque entre los hombres fueron más comunes las agresiones físicas. La violencia se produjo con mayor frecuencia en el hogar (37.6 %); sin embargo, los hombres fueron agredidos principalmente en la vía pública (42.4 %), comparado con las mujeres (30.7 %). También hubo diferencias en los factores de riesgo para sufrir violencia: en hombres se asocia con menor edad (60 a 64 años), mayor escolaridad (secundaria o más) y mayor nivel socioeconómico; en las mujeres, con tener depresión, no ser jefe de familia y la región del país.

**Conclusiones:**

La violencia contra las personas mayores se presenta de manera diferencial entre hombres y mujeres. Es necesario profundizar en los determinantes sociales de la violencia, en particular en el papel de la escolaridad entre los varones. La prevalencia relativamente baja, en comparación con otros estudios, puede obedecer a la dificultad y al temor para hablar sobre este tema tanto por razones culturales, como por la indefensión frente al escaso acceso a programas sociales que den protección y solución y que incidan sobre los determinantes sociales asociados a la violencia familiar y social que sufre este grupo poblacional.

## Background

Violence and abuse have harmful effects on the health and wellbeing of individuals, families, communities and countries [1]. The contribution of violence to the mortality and morbidity burden demands an increased investment of economic, social, and human resources, as well as a redefining of health priorities. The World Health Organization [2] defines violence as the intentional use of power, whether this comes in the shape of a threat or action against oneself, another person or a group, or community which leads to a high likelihood of injury, death or deprivation. When it comes to older adults, this can take place at the community level, when it is defined as violence, or be carried out by family or caretakers that reside within the home, where it is known as abuse [3, 4]

Current epidemiologic and demographic shifts have contributed to increasing the population of older adults, and this trend is expected to almost double from 9.7 in 2014 to 21.5 % by 2050 [5]. The decrease of family support options, which is a product of the changes in family structure and composition, are combined with the socioeconomic and health conditions that older adults may live with as well as changes in cultural values related to age. These act as determinants of violence and abuse towards older adults. Some international studies report that there is an association between these determinants and the deteriorating health status of older adults, which may include depression, advanced age, gender (female), higher education, low SES, social isolation, history of family violence, co-dependency with the aggressor, and characteristics of the primary care giver and their level of exhaustion [4, 6–8].

Studies about the prevalence of violence and abuse of older adults, are scarce and variable. In a systematic review of the prevalence of abuse in various countries, authors reported a wide variability of prevalence, from 3.2 to 27.5 %. This can be explained by the diversity of definitions, typologies, methodologies and instruments used [9, 10]. In a study conducted in Latin America, authors found that lifetime experience of physical violence towards older adults perpetrated by the spouse, was more frequent among women as compared to men, with a prevalence rate between 1.6 and 2.1 % [4, 11].

In Mexico, a study of four states found 16 % prevalence of abuse towards older adults, based on self-reported data [12]. Moreover, the National Family Life Survey, 2011, [13] reported the following prevalence for type of abuse for female older adults: 13.4 % emotional, 10.8 % neglect and 1 % physical abuse. In order to make the problem more visible and generate policies and interventions that can decrease violence towards this population, it is critical to understand the magnitude of the problem and the associated factors [7, 14, 15]. With respect to mortality due to violence among older adults, in 2013 it caused 4.2 % of all deaths among all older adults. This rate rose to 5.6 % among the 60–64 age group and was found predominantly among men (8.1 %) [3, 16]. The objective of the present study was to estimate the prevalence of violence towards older adults, as well as the associated factors, using data from Mexico’s 2012 National Health and Nutrition Survey, NHNS (ENSANUT in Spanish) [17].

## Methods

We conducted a secondary data analysis using data from Mexico’s 2012 NHNS, which is a probabilistic survey with multistage and stratified sampling that allows for a representative sample of the population at the state and national level, as well as by urban and rural strata. The sample comprised of 8,894 adults who are 60 years of age or older. Due to the expanding factor calculated in the NHNS design, this sample is representative of 10,747,490 older adults. The response rate for completed surveys was 87 % [18].

Surveyors asked the older adults about episodes of violence such as being the victim of robbery, aggression, assault, abuse or other types of violence in the last 12 months. For the positive responses to these questions, an initial analysis was conducted to describe the variables of violence (by unknown party) and abuse (by member of the family). However, given the low frequency of older adults who reported abuse and the difficulty conducting a rigorous statistical analysis, we decided to group the two variables together, creating the dependent variable (binary) labeled “violence/abuse”. The independent variables included in analysis were: age, sex, living with five or more people in the same house, head of household’s condition, education, having a partner, diagnosed with a non-communicable chronic disease (diabetes, hypertension, cardiovascular disease, other heart disease, or cancer), being diagnosed with depression by a health professional, having limited function (at least one limitation to daily activities such as walking, bathing, lying down/standing up, getting dressed or having a limitation in instrumental daily activities such as preparing food, buying food, taking medication, or managing money), cognitive deterioration (being unable to draw a clock, remembering 1 or 2, or no words), low self-esteem (self-classification as valuable individuals or not) and loss of power within the family (not consulted for important decisions or household finances or no contribution to household income). We also used region and socioeconomic status (SES) as an independent variable, and used the NHNS household and SES classification described by Gutiérrez and colleagues in 2013 [19] in line with the income deciles previously defined in the database, which corresponded to low (deciles 1 and 2), medium (deciles 3 to 6) and high (deciles 7 to 10). Finally, the country was divided into four geographic regions: North, Central-Western, Central and South-Southeast

We conducted a descriptive analysis of the variables with confidence intervals of 95 %. Considering the sampling strategy utilized for the survey permitted unbiased estimates. To calculate the variance, we used the mean total score for strata with a single sampling unit. The sample size varied based on the analysis variable. For the analysis of the variables of most interest with the population of older adults, using both men and women, we utilized the STATA command “subpop” (subpopulation).

We also evaluated the association of independent variables with the violence/abuse variable by using the chi-squared test or Fisher’s exact test, and crude odds ratios in the bivariate analysis. Finally, we used multiple logistic regression models to find adjusted estimates. This was done because of the complex survey design used for the NHNS; one for men and another for women. All statistical analysis was performed using STATA version 13.0 [20].

## Results

We analyzed data from 8,894 older adults (4,042 men and 4,852 women) with a mean age of 70.6 years. The prevalence of violence found was 1.7 % (CI 95 % 1.2 % to 2.2 %) [*n* = 119, *N* = 184,757], with no statistically significant different between men 1.7 % (CI 95 % 1.1 to 2.6) and women 1.7 % (CI 95 % 1.0 to 2.7).

Of the sample, a 119 older adults, 95.7 % (CI 95 % 91.7 to 99.6), reported experiencing violence, with a significantly greater proportion (*p* < 0.05) among men 99.4 % (CI 95 % 97.4 to 99.9 %), than among women 92.6 % (CI 95 % 81.3 to 97.3 %). Moreover, 4.3 % (CI 95 % 0.4 to 8.3) of the older adults reported to have been subjected to abuse, with a greater proportion among women 7.4 % (CI 95 % 2.7 to 18.7 %) than among men 0.6 % (CI 95 % 0.1 to 2.6 %).

In the violence/abuse category there were statistically significant differences between men and women by type of aggression (Table 1). In general, verbal aggressions dominated (62.9 %), followed by physical aggression in the form of “hitting, kicking, and punching” among men (32.3 %), and “other types of aggression or abuse” among women (18.7 %).

**Table 1:** Violence/abuse, prevalence of types of aggression among older adults, by sex. Mexico’s 2012, National Health and Nutrition Survey

	Men	Women	All
	*n* = 55	*n* = 64	*n* = 119
Type of aggression	% (CI 95 %)	% (CI 95 %)	% (CI 95 %)
Verbal aggressions	61.2 (41.9–78.2)	64.3 (44.1–88.4)	62.9 (48.7–75.1)
Hitting, kicking, punching	32.3 (16.4–56.7)	9.8 (4.3–20.6)	20.1 (11.7–32.3)*
Other aggressions or abuse	1.2 (0.3–4.6)	18.7 (7.4–39.8)	10.6 (4.4–23.4)*
Other	18.3 (8.3–35.7)	8.5 (3.8–17.7)	13.0 (7.4–21.9)
Pushing downward from an elevated location	1.8 (0.3–9.2)	2.1 (0.6–7.7)	2.0 (0.7–5.3)
Aggressions with substances	2.0 (0.3–13.4)	0.0-	0.9 (0.1–6.4)
Suffocation, strangulation, drowning	1.5 (0.2–10.1)	0.3 (0.004–2.4)	0.9 (0.2–4.3)
Sharp object wound (knife, blade, etc.)	0.1 (0.008–1.0)	1.4 (0.2–9.2)	0.8 (0.1–4.8)
Sexual assault	-	1.5 (0.3–7.2)	0.8 (0.2–3.9)
Poisoning or airway obstruction with substances or hot objects	-	-	
Firearm wound	-	-	-
NA/DK^a^	1.0 (0.2–7.0)	1.3 (0.2–8.9)	1.2 (0.3–4.7)

*p < 0.05

^a^NA/DK means Not answered/Don’t know

**Graph 1:**
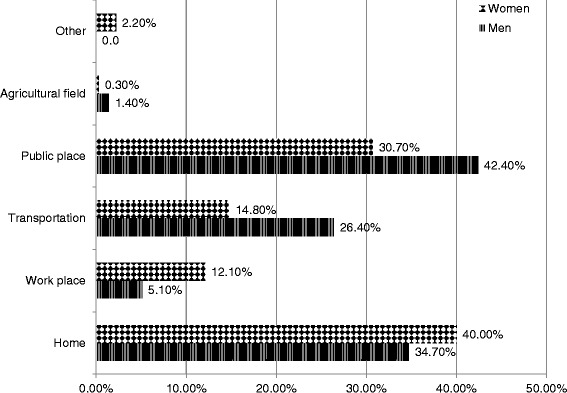
Prevalence by location where violence/abuse among older adults occurred. Mexico’s 2012, National Health and Nutrition Survey

With regards to where violence/abuse occurred, 37.6 % (CI 95 % 26.3 to 50.4) took place in the home, followed by 31.5 % (CI 95 % 20.3 to 45.3) of instances that took place in public spaces. The findings indicate that women experienced aggression or violence with greater frequency within the home at 40.0 % (CI 95 % 26.0 to 55.7), compared to men at 34.7 % (CI 95 % 18.0 to 56.2). In the case of the men, they experienced greater aggression in public places at 42.4 % (CI 95 % 17.1 to 52.7) than women at 30.7 % (CI 95 % 16.2 to 50.3) (Graph 1).

Regarding the care received by older adults following an aggression, 61.5 % (CI 95 % 48.0 to 75.4) were cared for by a family member, friend or neighbor, 26.1 % (CI 95 % 14.7 to 37.6) saw a chiropractor and only 0.6 % (CI 95 % -0.6 to 1.7) were seen by a medical professional.

In the bivariate analysis for men (Table 2), the factors associated with violence/abuse were age, education, and SES. Nearly 60 % of the older adults between 60 and 65 years of age were the victims of violence/abuse and were five times as likely to be victims (OR 5.7, CI 95 % 1.8 to 17.8) when compared to men 75 years or older. Over 50 % of those who had experienced violence/abuse has finished at least secondary school and were 2.6 times as likely to be victims (OR 3.6,CI 95 % 1.5 a 8.7), compared to men with less education. Lastly, 62.1 % of men who reported violence/abuse belonged to a high SES, with twice the likelihood of being a victim (OR 2.9, CI 95 % 1.1 a 7.3), compared to men with low SES. In the bivariate analysis for women (Table 3) nearly 42 % of the women who reported violence/abuse also reported depression and were three times as likely to be victims (OR 3.3, CI 95 % 1.5 a 7.0). The variables related to other diagnoses among older adults were not found to be statistically significant.

**Table 2:** Factors associated with the presence of violence abuse among older adult males. Mexico’s 2012, National Health and Nutrition Survey

Violence/abuse *n* = 4,042				
	No (*n* = 3,987)	Yes (*n* = 55)		
	% (CI 95 %)	% (CI 95 %)	OR (CI 95 %)	*P*-value
Age				
< 65 years	29.4 (27.1–31.8)	58.1 (37.8–76.0)	5.7 (1.8–17.8)	0.003
65–74 years	41.8 (39.2–44.5)	31.8 (16.5–50.5)	2.2 (0.8–6.1)	0.13
75+ years	28.8 (26.3–31–4)	10.0 (3.8–23.8)	1.0	
Lives with 5+ people	34.6 (32.1–37.1)	35.5 (19.4–55.6)	1.0 (0.4–2.4)	0.92
Is not the head of the household	12.8 (11.1–14.7)	8.8 (2.9–24.1)	0.7 (0.2–2.2)	0.49
Education				
Secondary+	23.9 (21.1–26.8)	53.3 (32.6–73.0)	3.6 (1.5–8.7)	0.003
Has partner	76.2 (74.0–78.2)	84.8 (69.5–93.2)	1.7 (0.7–4.3)	0.23
Region				
North	19.2 (17.4–21.1)	19.5 (9.2–36.7)	1.0	
North–west	24.5 (22.3–26.9)	20.4 (9.9–37.5)	0.8 (0.3–2.2)	0.69
Central	31.9 (28.9–35.1)	40.0 (19.2–65.1)	1.2 (0.4–4.1)	0.73
South-southeast	24.4 (22.4–26.6)	20.2 (9.2–38.7)	0.8 (0.3 (2.2)	0.69
SES				
Low	25.2 (23.2–27.4)	16.6 (7.7–32.2)	1.0	
Medium	37.1 (34.6–39.8)	21.2 (10.6–38.1)	1.2 (0.5–2.9)	0.76
High	37.7 (35.1–40.3)	62.1 (42.2–78.7)	2.9 (1.1–7.3)	0.03
Chronic disease	48.0 (45.5–50.6)	56.5 (34.0–76.6)	1.4 (0.6–3.6)	0.47
Depression	7.2 (5.6–9.2)	10.4 (2.2–37.9)	1.5 (0.3–8.1)	0.64
Functional dependence	44.1 (41.4–47.0)	25.9 (13.4–44.0)	0.4 (0.2–1.0)	0.05
Cognitive deterioration	18.6 (16.6–20.7)	18.3 (8.1–36.4)	1.0 (0.4–2.5)	0.98
Low self esteem	9.6 (8.1–11.3)	0.0	1.0	0.20
Dissatisfaction with life	2.7 (2.0–4.0)	2.7 (0.4–14.7)	1.0 (0.2–6.4)	0.98
Loss of power in the home	9.6 (7.9–11.7)	6.0 (1.8–17.8)	0.6 (0.2–2.1)	0.42
No economic contribution	9.5 (7.9–11.4)	6.9 (2.3–18.9)	0.7 (0.2–2.2)	0.55

**Table 3:** Factors associated with the presence of violence abuse among older adult females. Mexico’s 2012, National Health and Nutrition Survey

Violence/abuse *n* = 4,852				
	No (*n* = 4,788)	Yes (*n* = 64)		
	% (CI 95 %)	% (CI 95 %)	OR (CI 95 %)	*p*-value
Age				
< 65 years	29.9 (27.7–32.2)	37.7 (18.8–61.3)	1.8 (0.6–4.7)	0.26
65–74 years	40.5 (38.2–42.9)	41.1 (23.1–61.8)	1.4 (0.6–3.1)	0.40
75+ years	29.5 (27.5–31.7)	21.2 (11.5–35.8)	1.0	
Lives with 5+ people	32.5 (30.2–34.9)	31.9 (15.1–55.4)	1.0 (0.4–2.6)	0.95
Is not the head of the household	58.0 (55.7–60.3)	71.0 (56.1–82.5)	1.8 (0.9–3.4)	0.09
Education				
Secondary+	16.6 (14.5–18.9)	14.5 (7.4–26.8)	0.9 (0.4–1.8)	0.69
Has partner	46.4 (44.0–48.8)	51.4 (34.6–68.0)	1.2 (0.6–2.5)	0.57
Region				
North	19.5 (17.7–21.3)	9.1 (4.2–18.5)	1.0	
North-west	23.1 (21.1–25.3)	28.2 (13.9–48.9)	2.6 (0.97–7.1)	0.06
Central	35.1 (32.4–38.0)	43.6 (24.1–65.3)	2.7 (0.95–7.5)	0.06
South-southeast	22.3 (20.3–24.4)	19.1 (4.2–18.5)	1.8 (0.6–5.3)	0.25
SES				
Low	25.5 (23.4–27.7)	14.6 (7.0–28.1)	0.5 (0.2–1.3)	0.17
Medium	41.2 (38.6–43.8)	45.8 (27.9–64.9)	1.0	
High	33.3 (30.8–35.9)	39.6 (23.2–58.7)	1.1 (0.5–2.5)	0.88
Chronic disease	60.7 (58.3–63.0)	59.9 (41.1–76.2)	1.0 (0.4–2.1)	0.94
Depression	17.9 (16.2–19.8)	41.6 (25.3–60.0)	3.3 (1.5–7.0)	0.003
Functional dependence	33.7 (31.4–36.0)	30.7 (16.4–49.9)	0.9 (0.4–2.0)	0.74
Cognitive deterioration	24.6 (22.6–26.7)	13.7 (6.7–25.9)	0.2 (0.2–1.1)	0.08
Low self esteem	7.8 (6.5–9.4)	7.0 (2.4–18.4)	0.9 (0.3–2.7)	0.81
Dissatisfaction with life	3.1 (2.3–4.1)	1.2 (0.2–8.3)	0.4 (0.05–2.9)	0.35
Loss of power in the home	14.3 (12.7–16.0)	25.8 (9.7–53.1)	2.1 (0.6–6.9)	0.23
No economic contribution	36.7 (34.2–39.3)	21.3 (9.0–42.5)	0.5 (0.2–1.3)	0.14

In the multiple regression models (Table 4), the factors associated with violence/abuse (adjusted OR) were different for men than for women. For men, age was the most important factor, with nearly four times the likelihood of suffering violence/abuse in the 60–65 years-of-age range and the highest education. In women, depression increased the likelihood of experiencing violence/abuse by 3.4 times, more so than other factors such as not being the head of the household or region of the country. The North region serves as a reference, while women in the Central region of the country had greater likelihood of undergoing violence/abuse, and women in the South-Southeast and Northwest, less.

**Table 4:** Summary of factors associated with violence/abuse among older adults, compared by sex. Mexico’s 2012, National Health and Nutrition Survey

Men		Women	
*n* = 4,042		*n* = 4,852	
	Adj. OR (CI 95 %)^a^		Adj. OR (CI 95 %)^b^
Age		Not the head of the household	2.9 (1.1–7.7)
< 65 years	3.9 (1.7–9.0)	Region	
65–74 years	1.6 (0.6–4.5)	North	
75+ years	1.0	North-west	3.8 (1.1–13.2)
Education		Central	3.6 (1.0–12.5)
Secondary or higher	2.6 (1.4–5.0)	South-southeast	2.7 (0.8–8.9)
		Depression	3.4 (1.4–8.4)

^a^Separate models for men (Goodness-of-fit test, *p* = 0.33)

^b^Separate models for women (Goodness-of-fit test, *p* = 0.06)

## Discussion

This study represents a contribution to the identification of the magnitude of violence and abuse experienced by older adults at the national level, especially taking into account that the extant literature is often restricted to the regional or local level, or to specific cases or violence in the home [4, 11, 21, 22]. In contrast, our data set allowed us to look at violence at the national level. We found that violence/abuse towards older adults in Mexico can be perpetrated by both unknown and known members of the family alike, with the latter being dominant.

It is critical to pay attention to the needs of the older adult population in terms of public policy, even more when we take into account the context of social violence in most Latin American countries, and the negative changes in values towards older adults [23, 24]. In Mexico, the magnitude and dynamic of this population group presents particular characteristics. About 9.12 % of the population is 60 years of age or older, and there is at least one older adult currently residing in 39 % of the homes. Moreover, the population is growing faster than any other group in the country, at 3.5 % annually. That is double the rate of growth of the total population of the country [5].

The dominant finding of violence perpetrated by unknown parties and the high percentage of incidents occurring outside the home (close to 60 %) and primarily among men, has implications for both social and health policy. In particular, policies must address the double effect experienced by older adults: social violence that is occurring throughout the country, and which produces fear and social isolation [25], and also violence that comes from abuse perpetrated by a family member. This multidimensionality calls for more in-depth research and analysis of what occurs in the different spaces in which older adults live their lives, so that the primary determinants of this abuse can be clearly identified and be used for designing prevention programs with an intersectoral focus.

As with other studies [23, 26–28], gender seems to be the most important determinant to describe the characteristics of violence/abuse in Mexico, given that the settings and types of aggression occurs in relation to the social roles traditionally assigned to men and women, respectively. Men presented more physical aggressions than women, and their abuse mostly took place outside the home at the hands of an unknown party. In turn, women experienced more psychological violence and this often occurred inside the home, perpetrated by a family member. These differences are associated with the greater presence of men in the public sphere, as well with men’s traditionally higher economic independence. Finally, this abuse may also be related to risky attitudes tied to the masculine social role. In contrast, older female adults spend more time in the home, and a great percentage of them have to rely on the economic help from family members [11, 29].

The logistic regression models also indicate that factors associated with violence are divergent by gender. For men, the factors include lower age (60 to 64 years), supported by findings from Cuban studies demonstrating that older adults within the 60–69 age range were more likely to experience psychological and social violence, [30] and that this correlates with being more educated (secondary school or higher) and higher SES [31]. For women, the factors include having depression, not being the head of the household, and place of residence by region of the country.

Although psychological abuse was more dominant than physical abuse, the reality of one in every five older adults reporting being physically abused requires further investigation. The underreporting of this type of adverse event may be significant, given the fear or shame that may exist in reporting [15, 32, 33].

International studies found that having a partner, spending time with family, and suffering a chronic disease are all factors associated with violence and abuse [28, 34–37]. However, these were not statistically significant findings in our study. Moreover, a factor mentioned in other studies that was not included in the NHNS was a history of family violence [4, 11, 23]. In regards to episodes of violence or abuse in which the victim sought care, they reported seeking help at home or with a chiropractor. The low utilization of health services in terms of help seeking in cases of violence identified in previous studies [38] was also a key finding for this study. This may, in part, explain the low prevalence of violence found through the health system’s reporting mechanisms. It is necessary to take a more in depth look at the factors associated with the underutilization of health services following an event of violence or abuse.

Although the problem of violence among older adults has been characterized for its intersections in age, gender, and poverty, in this survey SES was not a significant factor for either men or women. In fact, men with higher education and higher SES reported great rate of violence perpetrated by unknown parties. This was an unexpected result. However, these findings are in line with others conducted among Latin American populations, and has often been explained by characteristics related to values inherent to masculinities in the region, the gender roles, and the level of empowerment fostered by education and income, which may lead to better reporting of abuse [8, 31]. Further studies that can provide more understanding on this finding are required.

Violence is an issue of power relations among persons, and less power may lead to an increased risk for abuse. This may explain why women who were not the head of their households faced greater probabilities of being assaulted and less probability in reporting the incident [39]. This dependency may generate stress and depression, which are factors leading to increased abuse, especially within the context of poverty. The results from Mexico’s 2012 NHNS are in line with other studies demonstrating depression as a risk factor for abuse among the older adult population; this particular morbidity is highly prevalent among women in Mexico, resulting in vulnerability of abuse [6, 7, 28, 40, 41].

This study also reflects the limitations posed by the NHNS. For example, the underreporting of violence perpetrated by a family member are framed by the consequences that the older adult that responded to the survey may face. We must also consider the very limited access to social programs that provide a solution and that intervene at the levels of determinants of social and family violence that impact this population group [9, 42]. Moreover, the cross-sectional analysis may have residual confounding effects due to variables not included, given that the NHNS used a general questionnaire as opposed to an instrument that was designed specifically to detect violence among older adults. Another limitation for the NSNH survey is that the category ‘other types of aggression or abuse’ does not discern or unpack its content.

## Conclusions

The Mexico’s 2012 National Health and Nutrition Survey allowed estimating the prevalence of violence towards older adults, as well as the associated factors.

Gender, and its implications in terms of education, income, social status, empowerment and self-esteem seem to explain the differences in the type, frequency and place of violence in older adults. In this regard, it is necessary to increase knowledge about the dynamics of the social determinants of violence, in particular the role of education and SES among men.

The complexity of the problem and gaps in the available information warrant further research that can explore this issue through the use of diverse methodologies and that include a consideration of the sociocultural, economic, and health system context. We also suggest a focus on the social determinants, both in the national and regional sphere, which takes into account the international agreements that exist to reduce the victimization of older adults and ensure their rights and dignity [42]. We highlight the need for public policies and community strategies aimed to abuse and violence prevention for older adults that are built on a culture of respect for human rights, and the participation of multiple stakeholders [23, 27] in the social process of enhancing the wellbeing of older people in the family and society.
